# Resident Quality of Life and Family Satisfaction: Developing Measures for Minnesota Assisted Living Report Card

**DOI:** 10.1093/geroni/igaa057.2579

**Published:** 2020-12-16

**Authors:** Muriel Wheatley, Valerie Cooke, Tetyana Shippee

**Affiliations:** 1 Vital Research, Los Angeles, California, United States; 2 Minnesota Department of Human Services, St. Paul, Minnesota, United States; 3 University of Minnesota, Minneapolis, Minnesota, United States

## Abstract

The 2019 Minnesota Legislature requested the Department of Human Services (DHS) and Minnesota Board on Aging to develop and administer a report card for assisted living (AL), including conducting annual resident and family surveys in Minnesota AL settings. This presentation includes the perspectives of representatives from MN DHS and Vital Research, as well as the University of Minnesota team who worked together to develop survey items, carry out the cognitive testing, and conduct analyses. Survey items were developed from published literature and existing tools on assisted living quality and underwent testing with MN stakeholders and cognitive testing with MN AL residents. Pilot testing assessed any further changes that needed to take place for resident and family satisfaction with AL quality (n=400). Presenters will share lessons learned with implementing the new tools and different aspects of the of the report card development and implementation process as well as the survey findings.

